# Exploring infectious disease spread as a function of seasonal and pandemic-induced changes in human mobility

**DOI:** 10.3389/fpubh.2024.1410824

**Published:** 2024-08-27

**Authors:** Ruiqing Cai, Zach Spencer, Nick Ruktanonchai

**Affiliations:** ^1^Johns Hopkins Bloomberg School of Public Health, Baltimore, MD, United States; ^2^University of Pittsburgh School of Public Health, Pittsburgh, PA, United States; ^3^Population Health Sciences, Virginia Tech, Blacksburg, VA, United States

**Keywords:** infectious disease epidemiology, human mobility, mathematical modeling, outbreaks, respiratory disease

## Abstract

**Introduction:**

Community-level changes in population mobility can dramatically change the trajectory of any directly-transmitted infectious disease, by modifying where and between whom contact occurs. This was highlighted throughout the COVID-19 pandemic, where community response and nonpharmaceutical interventions changed the trajectory of SARS-CoV-2 spread, sometimes in unpredictable ways. Population-level changes in mobility also occur seasonally and during other significant events, such as hurricanes or earthquakes. To effectively predict the spread of future emerging directly-transmitted diseases, we should better understand how the spatial spread of infectious disease changes seasonally, and when communities are actively responding to local disease outbreaks and travel restrictions.

**Methods:**

Here, we use population mobility data from Virginia spanning Aug 2019-March 2023 to simulate the spread of a hypothetical directly-transmitted disease under the population mobility patterns from various months. By comparing the spread of disease based on where the outbreak begins and the mobility patterns used, we determine the highest-risk areas and periods, and elucidate how seasonal and pandemic-era mobility patterns could change the trajectory of disease transmission.

**Results and discussion:**

Through this analysis, we determine that while urban areas were at highest risk pre-pandemic, the heterogeneous nature of community response induced by SARS-CoV-2 cases meant that when outbreaks were occurring across Virginia, rural areas became relatively higher risk. Further, the months of September and January led to counties with large student populations to become particularly at risk, as population flows in and out of these counties were greatly increased with students returning to school.

## 1 Introduction

The COVID-19 pandemic has transitioned to a long-term management period, particularly with the World Health Organization declaring that the COVID-19 pandemic no longer constitutes a public health emergency of international concern (PHEIC) (1). This comes after a period of wide ranging impacts that dramatically changed how people interact and consider infectious disease risk (2–4). As the global spread of future emerging diseases remains a critical concern, the COVID-19 pandemic provides many valuable lessons for how future outbreaks will spread, and how communities may respond to future emerging diseases.

In particular, the pandemic highlighted how significant changes to mobility can dramatically change connectivity networks more generally (5, 6), affecting infectious disease spread in unexpected ways (7). Most notably, non-pharmaceutical interventions changed the spatial dynamics of SARS-CoV-2 over time because some communities strictly adhered to stay-at-home measures and other interventions, while others quickly exhibited pandemic fatigue and began returning to normal travel and contact patterns (8, 9). This led to “waves” that became difficult to control both internationally and domestically, as outbreaks moved between communities that experienced reduced intervention adherence. These community-level differences in intervention adherence have previously been explained based on community-level factors such as income, political beliefs, perceptions of legitimacy (10), and urbanization (11). The resulting landscape of intervention adherence then led to unexpected differences in long-term COVID-19 risk and mortality for the communities that did not adhere as strongly (12, 13), and influenced new waves of COVID-19 cases (14), as outbreaks occurred in places that were no longer reducing their travel patterns and spread to nearby areas.

Changes to community-level travel patterns and overall connectivity can occur outside of a global pandemic, as well (15–17). Seasonal, holiday, and catastrophe-related changes in travel patterns influence both local contact patterns and disease spread. Seasonal differences in travel have been previously observed both during and before the pandemic, where people will tend to visit green spaces and travel long distances more often during the summer (18). Beyond this, holidays have been shown to lead to large-scale population mobility changes that affect the spread of disease (19), even during the emergence of COVID-19 (20). Catastrophic events have also dramatically changed communities’ travel patterns and disease spread, such as during floods or earthquakes when mass displacement can cause new outbreaks of disease, particularly when local healthcare infrastructure is impaired (21, 22).

As the world experiences increasing social and demographic change and becomes more interconnected through global travel, it will be critical to understand how the emergence of future diseases will be impacted by both spontaneous and regular changes to connectivity and travel. Accurately predicting mobility and disease spread is critical for predicting at-risk areas and hotspots during any outbreak (23), and understanding how these processes may change during major events or seasonally is essential for future epidemics and emerging diseases. This is particularly relevant as many potential new human diseases are likely to be directly transmitted in nature (24), similar to SARS-CoV-2. For these diseases, interventions focused on human mobility will likely remain a front-line defense before effective treatments and vaccines are developed.

Here, we analyze novel high-resolution population mobility data from before and during the COVID-19 pandemic to better understand how mobility changed seasonally and during the pandemic, and how these changes could impact the potential spread of a newly emerging disease, particularly if NPIs such as lockdowns and stay-at-home orders are used again. To do this, we simulate the spread of a hypothetical emerging directly transmitted disease across Virginia using statewide mobility patterns from the pre-pandemic and during pandemic periods. We then determine the months and locations most likely to lead to widespread transmission, and focus on characterizing the differences in outbreak potential between urban and rural areas, both before and during the COVID-19 pandemic. We also explore other seasonal patterns, focusing on the importance of late summer and early months of the year to outbreak potential in certain counties. Overall, this work provides important insights into how the landscape of connectivity and expected disease spread changes seasonally and with the introduction of an emerging disease, and helps explain how urbanization drives some of these differences.

## 2 Methods

Using a stochastic discrete-time SEIR metapopulation model, we simulated the spread of a hypothetical directly transmitted human disease across various possible starting months and locations in Virginia, using daily-level mobility data flows obtained from SafeGraph. We analyzed how spread of the disease over the first 75 days of the outbreak depended on both the starting month and location, to identify the places and periods most likely to lead to epidemic spread, and how these two factors interacted across time and space.

### 2.1 Data

The mobility data for this study was collected from the SafeGraph database, which contains anonymized cell phone location data for millions of devices across the United States (25). The data was collected for the period from August 1, 2019 to March 1, 2021 for the state of Virginia. This dataset provided daily origin–destination matrices at the county-level across Virginia, representing travel across all 133 counties and independent cities. We took the average of all matrices for each month, to aggregate this dataset and obtain monthly population mobility patterns. This ultimately led to 19 adjacency matrices that were 133 × 133 in size, where element $\left[i,j\right]$ indicates the number of people who traveled per day from county i to county j, on an average day for each of 19 months. The resulting monthly mobility matrices reflected the average number of people who moved from each county to each other county, on an average day during that month. This data also provided an estimate of total population in each county, which allowed us to calculate the fraction of people traveling from each county to each other per day. For each month, we used this data to inform movement across patches and the relative contact rate for each county.

### 2.2 Simulation model

To simulate the spread of infectious disease using mobility patterns from various months, we used an SEIR model that incorporates mobility through moving people between patches at the end of each simulation step. This model has been used previously to model the spread of COVID-19 (26). In this model, once people recover from the disease, they then gain immunity to the disease. This is likely not the case for many diseases, including COVID-19, but was suitable as we only sought to capture the dynamics of spread during the initial outbreak, rather than analyze a potential steady state. Accordingly, we simulated the spread of disease for 75 days after the initial infection.

The infection component of the model is represented by the following equations:


$$\frac{dS_{i}}{dt}=-\frac{\beta_{i,t}S_{i}I_{i}}{N_{i}}$$

$$\frac{dE_{i}}{dt}=\frac{\beta_{i,t}S_{i}I_{i}}{N_{i}}-\varepsilon E_{i}$$

$$\frac{dI_{i}}{dt}=\varepsilon E_{i}-\gamma I_{i}$$

$$\frac{dR_{i}}{dt}=\gamma I_{i}$$

where $\beta_{i,t}$ represents a patch-and month-specific transmission rate, ε represents the rate of becoming infectious, and γ represents the recovery rate for the disease. $\beta_{i,t}$ varied across counties with the month used to parameterize mobility for a given simulation run. This value reflected the relative number of travelers who moved out of a county compared to the average number of trips out of that county per day across pre-pandemic months (August 2019 to February 2020). If the pre-pandemic average number of trips out of a county i was $\bar{n_{i}}$ , and the number made out in the month of simulation was $n_{i,t}$ , then


$$\beta_{i,t}=\beta \left(1+\frac{n_{i,t}}{\bar{n_{i}},}\right)/2$$

Where β=0.6 (see Table 1). This reflects the previous observation that reductions in longer-distance trips correlated strongly with contact rates across the pandemic [cite]. We opted for this formulation of $\beta_{i,t}$ to make the minimum transmission rate possible 50% of the original transmission rate, reflecting that some contacts cannot be avoided, even when travel is reduced significantly. $\beta_{i,t}$ was the only parameter that varied across simulation runs. Other parameter values used are described in Table 1. We used values reflecting a disease with an incubation period of 5 days, an infectious period of 10 days, and a reproductive number of 2.68, an estimate obtained from initial studies of SARS-CoV-2 in China (27).

**Table 1:** Parameter values used in this study.

Parameter	Biological interpretation	Starting value
β	Per capita transmission rate	0.268
ε	Rate of becoming infectious	0.2
γ	Rate of recovering with immunity	0.1

Using this model, we simulated the spread of a disease in discrete-time, where infection-related processes occur, then movement between patches occurs as a separate step. County-to-county mobility was parameterized using the SafeGraph data, where the daily probability of moving from patch i to patch j was quantified as the fraction of people who moved from i to j on average, per day, for the month simulated. Each simulation run also varied based on the $\beta_{i,t}$ values used, which depended on the month simulated t . Finally, we varied the county where the initial 10 infections were located, to observe the potential spread of disease out from each location.

Our simulations were run for 75 days; because the mobility networks and $\beta_{i,t}$ values did not change within a single simulation, each simulation reflects how the disease would have spread over 2.5 months if mobility patterns did not change over that period. For example, if the initially infected people were in Montgomery County and we used mobility data from January 2020, that simulation run would reflect how a disease would spread out of Montgomery County if emergence occurred in January 2020 and mobility patterns did not change over the following 75 days.

We ran 100 simulations of each combination of the monthly mobility matrix and starting location for 75 days each, yielding 226,000 total simulation runs. The simulation model was programmed using R, and a version of this model has been used previously to understand the spread of COVID-19 and is available online on GitHub.^1^

### 2.3 Contextualizing model outputs

In our initial simulation runs, we found that urban areas led to more spread, particularly if emergence occurred during pre-pandemic months. We therefore investigated how urban communities responded to COVID-19. We hypothesized that urban areas would be much more likely to maintain mobility restrictions and return to normalcy much more slowly than rural areas, which has been observed previously (28). To test this, we used data from the United States Census, which matched counties with a delineation of metropolitan or micropolitan based on their population density.

Once we matched each county and city with a statistical area classification, we quantified the change in mobility for each area for the 12 months following March 2020, compared to the corresponding month a year earlier. We then used Welch’s two sample *T*-tests to compare mobility in metropolitan and rural areas for each month against their pre-pandemic means.

## 3 Results

**Figure 1:**
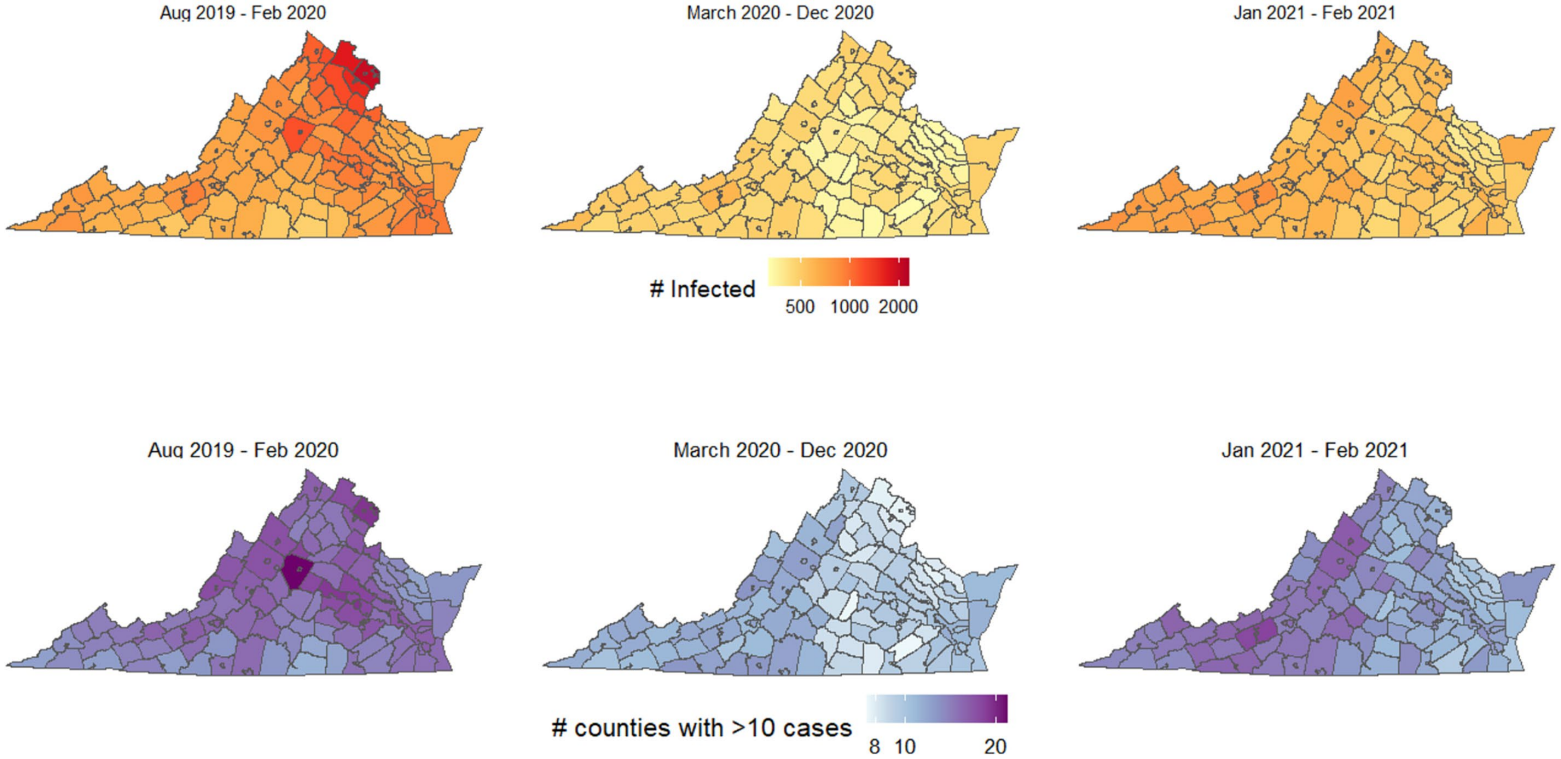
Outbreak potential of each county, using mobility patterns from before the COVID-19 pandemic began, the first 9 months of the pandemic (March–December 2020), and January–February 2021, after many interventions had been lifted. Top shows the number of people infected if an outbreak begins in each county, bottom shows the number of counties with at least 10 infected people if the outbreak begins in each county.

First, we simulated the spread of disease from each potential starting location, after averaging the mobility patterns for all “pre-pandemic” months of data (September 2019 to February 2020), for all “early stage pandemic” months of data (March 2020 to December 2020), and for “later stage pandemic” months of data (January 2021 and February 2021). Figure 1 shows the spread of disease for each starting location if using pre-pandemic human mobility patterns. This figure illustrates the outbreak potential for each county using mobility patterns from each of these three periods, where the shading for each county illustrates the number infected after 50 days if the outbreak begins there (top, Figure 1), or the number of counties with at least 10 infected people after 50 days (bottom, Figure 1).

**Figure 2:**
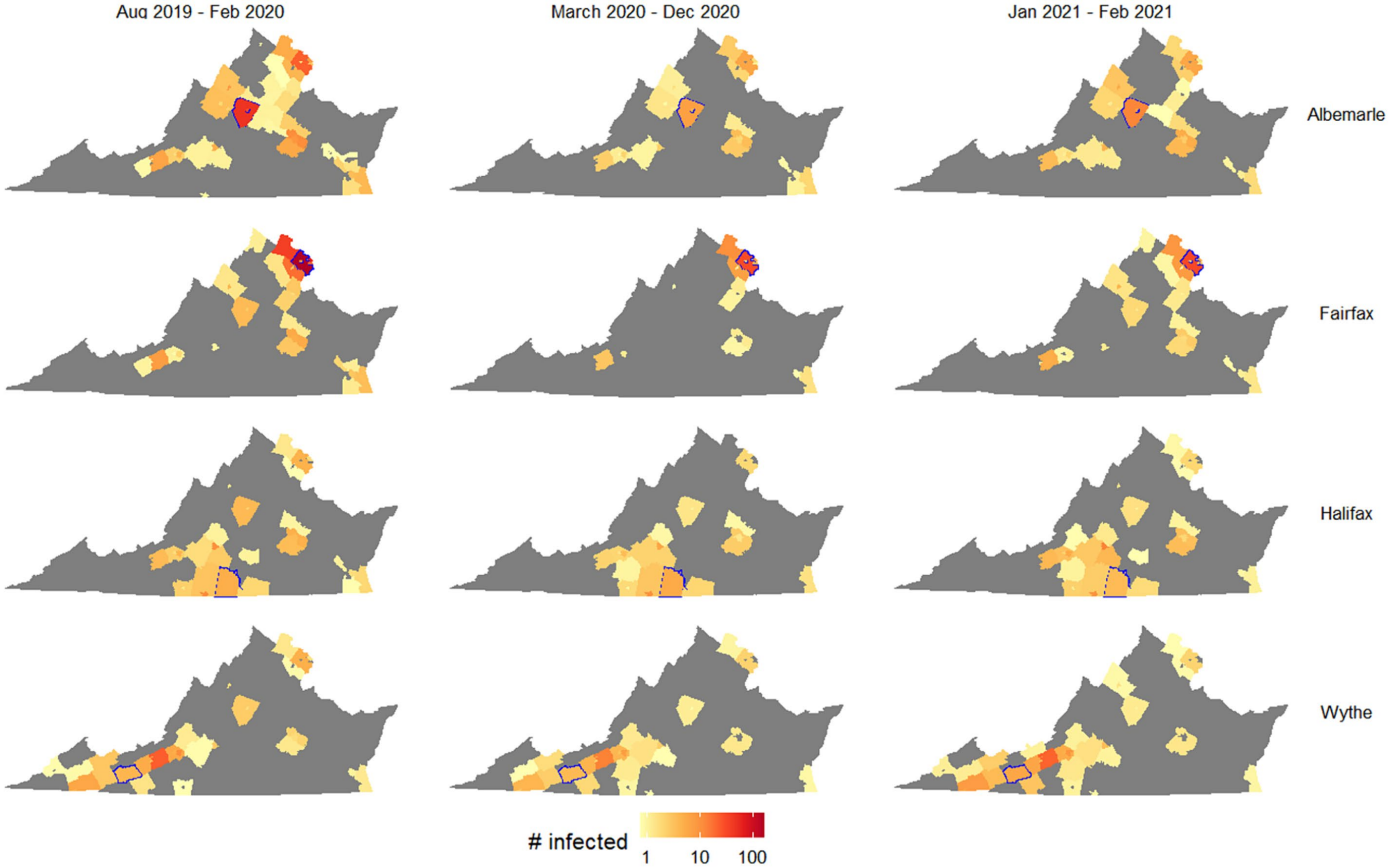
Outbreak potential of four target counties, measured as number of counties with at least one case after 50 days of simulation on average. Columns separate simulations by the mobility patterns used, and rows separate simulations by the starting county (highlighted in blue in each map).

The most spread occurred using mobility patterns from the pre-pandemic period, and key locations led to particularly high spread and infections, including the northern Virginia region and Charlottesville. Figure 2 shows how the spatial spread locations varied, for selected counties in northern Virginia, Charlottesville, Southside Virginia, and Southwest Virginia. The values and interquartile ranges associated with the results shown in Figures 1, 2 are included as Supplementary Table S1.

Figure 2 suggests that while outbreak size generally shrank when using pandemic-era mobility patterns, this decrease was much larger for counties with urban centers (Albermarle and Fairfax) than rural counties (Halifax and Wythe). Specifically, Fairfax was associated with a much smaller spatial spread of disease using pandemic-era mobility patterns, as starting an outbreak in this county resulted in 34 counties with infected people after 50 days using August 2019 to February 2020 mobility patterns (interquartile range 14–41), while half as many (17) had had infected people using March 2020 to December 2020 mobility patterns (IQR 5–32). In contrast, when outbreaks began in Wythe County, there were very small differences in the numbers of counties with infected people after 50 days (31, 28, and 33, respectively, for pre-, during, and late-stage pandemic mobility patterns; respective IQRs of 9–40, 7–39, and 10–49). This likely reflected the greater reduction in cross-county travel exhibited by urban areas in the first year of the pandemic, particularly as people chose to work from home.

**Figure 3:**
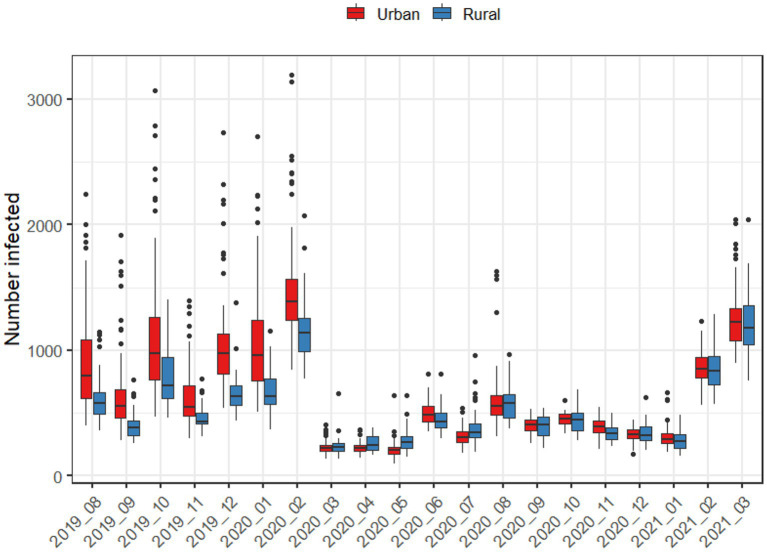
Average number of infections after 50 days, when an outbreak begins in either an urban or rural county, using the mobility patterns of each month.

Figure 3 further demonstrates the role of urbanization in driving disease spread both using pre-pandemic and pandemic-era mobility patterns. Using pre-pandemic mobility patterns, outbreaks in metropolitan areas led to larger numbers of infections, while using pandemic-era mobility patterns, metropolitan and non-metropolitan areas led to similar numbers of infections.

**Figure 4:**
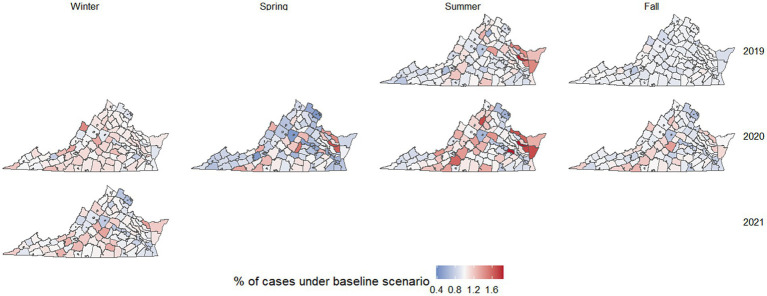
Heatmap of Virginia counties and cities associated with their change in mobility when comparing each season to the average of September 2019 to February 2020. Counties and cities with an increase in mobility are denoted by a shade of red while counties and cities with a decrease in mobility are denoted by a shade of blue. Shades of red and blue appear darker when values are further from one.

When directly comparing mobility in metropolitan and rural areas during the pandemic (Figure 3), metropolitan areas exhibited statistically significantly lower mobility across all pandemic-era months. For metropolitan areas, the largest decreases in mobility occurred in May 2020 and December 2020, while June and August 2020 reflected the point where mobility was the closest to normal. In every case, metropolitan areas had lower levels of mobility than rural areas. For rural areas, only April, August, September, and November were statistically significantly lower than the baseline. Aggregating mobility to seasons, there were spatial patterns in mobility reductions during pandemic-era months as well (Figure 4); for example, the Chesapeake Bay area had increased mobility during the summer months, and we also observed higher mobility in counties with large universities in the winter months.

We also performed initial sensitivity analyses, varying the value of β and the average probability of people moving per day. We found that changing these values either sped up or slowed down the spread of the outbreak, but did not change the spatial dynamics of the simulated outbreaks. If β was increased dramatically, there was a chance that the outbreak would spread rapidly within its initial patch and not spread to other patches, as people recovered with immunity and caused the outbreak to die out before spread could occur.

## 4 Discussion

By simulating the spread of an emerging respiratory disease using mobility patterns from different periods, our work emphasizes how connectivity networks are not static. They change when major events occur (such as during the COVID-19 pandemic, or floods and earthquakes) and seasonally, particularly during holiday periods. These changes to connectivity can also have dramatic impacts on the spread of any newly emerging disease. Most notably, the pre-pandemic and pandemic periods exhibited dramatically different networks of disease spread, demonstrating the effect of potential community response to disease spread. For example, sociocultural context caused certain counties and months to lead to unusually high levels of spread, such as counties with large research universities during January and September (the beginning of Spring and Fall semesters, generally).

We found that urbanization was one of the most important community-level distinctions that affected the spread of COVID-19 before and during the pandemic. Interestingly, pre-pandemic mobility patterns led to much faster disease spread out of urban areas than out of rural areas, both in terms of numbers of people infected and number of counties with significant outbreaks after 50 days (Figure 1). The faster pre-pandemic spread in urban areas reflects the higher population density of urbanized counties, and that urban communities generally move to neighboring areas more frequently than rural communities. This is exemplified by the commuter patterns of northern Virginia, the largest urban region of the state, where people regularly travel between counties for work, school, and commerce (Figure 2).

**Figure 5:**
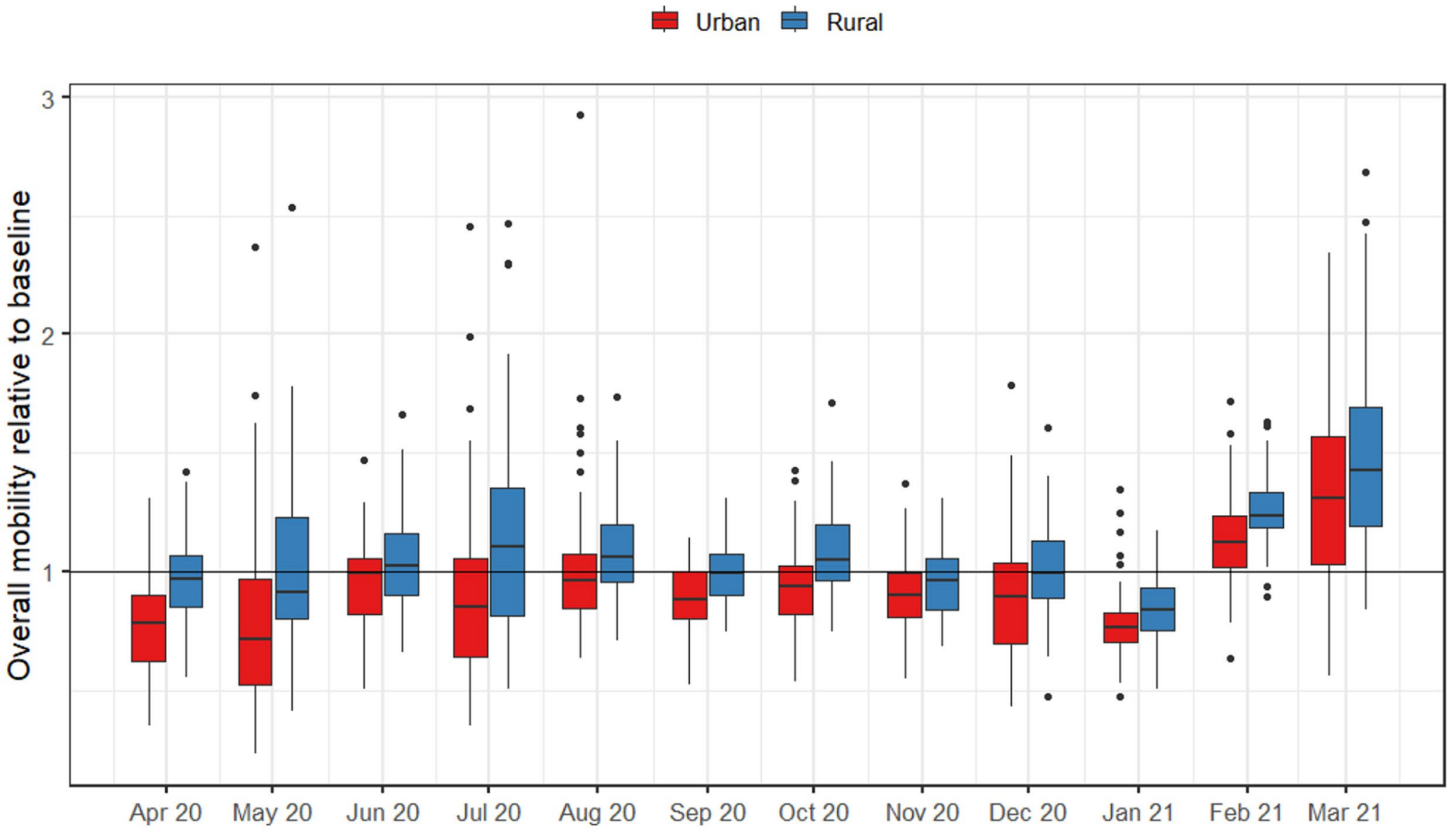
Overall numbers of trips outside of the county, by urban and rural counties throughout Virginia for months after March 2020. Baseline mobility was measured as the average monthly number of trips for August 2019 to February 2019.

During the pandemic months, travel patterns changed dramatically, and the discrepancy in mobility between urban and rural counties largely disappeared. Across all months starting March 2020, there was no longer any noticeable difference between urban and rural counties in terms of both total numbers of people infected and numbers of counties with a significant outbreak after 50 days (Figure 3). Our statistical analyses suggest that this is due to urban areas having categorically lower levels of mobility than rural ones throughout the pandemic, even during periods when restrictions were lifted (Figure 5).

Outside of the pre and during pandemic differences, we also found that certain hotspots of outbreak spread occurred in certain months, highlighting the increased risk when major events coincide with disease emergence. This includes counties with significant research universities, which led to high amounts of spread during months when students were traveling to/from school (Figure 4). Rural areas around Chesapeake Bay also experienced unusually high amounts of mobility and potential disease spread in May and July for both 2019 and 2020, which could be explained by holiday-related travel (Figure 4).

Our study importantly only examined the state of Virginia, while much of the USA and rest of the world experienced very different NPIs and have a different social and cultural context that could impact how communities respond, and how mobility networks change during major events. Additionally, our data on mobility were limited in terms of understanding the populations that travel, and how long people are away during travel. In future studies, accounting for the specific communities that are most likely to travel or stay home, and whether the characteristics of trips (such as duration and prophylactic behaviors undertaken during travel) change seasonally or during major events will be necessary to build on our work.

Overall, the amount of spread expected when a disease has an outbreak varies significantly with time and location. Unsurprisingly, the COVID-19 pandemic induced the largest changes to the overall transmission network, but seasonal changes meant some locations were likely to lead to especially high outbreak spread some months, based on key events such as school openings and summer holiday seasons. There were large differences in outbreak potential between counties as well, as some counties led to 5 times as many cases as the least-connected counties. While we describe these effects in the context of the pandemic, school sessions, and holiday seasons, there is still significant unexplained spatiotemporal variation in outbreak potential, and future work will be needed to better understand what factors drive local hotspots to occur. By identifying when and where local potential outbreak hotspots are, policymakers can better prepare for future emerging diseases and outbreaks as they occur.

## Data availability statement

The mobility data used in this study can be found from the University of Wisconsin Madison GeoDS lab website, https://github.com/GeoDS/COVID19USFlows.

## Ethics statement

The studies involving humans were approved by the Virginia Tech Institutional Review Board. The studies were conducted in accordance with the local legislation and institutional requirements. Written informed consent for participation was not required from the participants or the participants’ legal guardians/next of kin in accordance with the national legislation and institutional requirements.

## Author contributions

RC: Formal analysis, Validation, Writing – original draft, Writing – review & editing. ZS: Formal analysis, Validation, Writing – original draft, Writing – review & editing. NR: Conceptualization, Data curation, Formal analysis, Funding acquisition, Methodology, Project administration, Supervision, Visualization, Writing – original draft, Writing – review & editing.

## Footnotes

^1^ https://github.com/wpgp/BEARmod
